# Treatment-induced neuropathy of diabetes overlapping with chronic inflammatory demyelinating polyradiculoneuropathy after rapid glycemic correction: a case report

**DOI:** 10.3389/fendo.2025.1654872

**Published:** 2025-11-19

**Authors:** Yonglu Hu, Bingzi Dong, Lijun Liu, Yujie Deng, Runhong Han, Chengqian Li

**Affiliations:** 1 Department of Endocrinology and Metabolism, The Affiliated Hospital of Qingdao University, Qingdao, China; 2 Department of Neurology, The Affiliated Hospital of Qingdao University, Qingdao, China; 3 Department of Nephrology, The Affiliated Hospital of Qingdao University, Qingdao, China

**Keywords:** treatment-induced neuropathy of diabetes (TIND), chronic inflammatory demyelinating polyradiculoneuropathy (CIDP), neuropathy pain, T1DM, case report

## Abstract

Treatment-induced neuropathy of diabetes (TIND) often presents with acute-onset neurological symptoms and is frequently underdiagnosed in clinical practice, typically occurring after rapid correction of hyperglycemia. Its coexistence with chronic inflammatory demyelinating polyradiculoneuropathy (CIDP) has rarely been reported. As both conditions involve peripheral neuropathy and inflammation, they may share common underlying mechanisms. We report the case of a 26-year-old male patient with type 1 diabetes mellitus (T1DM) who developed TIND followed by CIDP. The patient received insulin pump therapy for glycemic control, along with oral analgesics, including gabapentin and carbamazepine. Targeted treatment for CIDP with methylprednisolone was initiated, leading to improvement in neurological symptoms. Due to persistent severe perineal pain, the patient also underwent interventional pain procedures, including perineal nerve pulsed radiofrequency therapy. Additionally, we review the literature on the pathophysiological mechanisms of TIND and discuss its potential relationship with CIDP, aiming to provide clinical insights that may assist in early recognition and diagnosis. To date, cases of coexisting TIND and CIDP in a single patient have rarely been reported in the literature.

## Introduction

Treatment-induced neuropathy of diabetes (TIND) is an iatrogenic complication associated with insulin therapy, particularly in patients with type 1 diabetes mellitus (T1DM) (1, 2). It typically emerges in chronic hyperglycemia, often following a rapid reduction in HbA1c levels due to improved glycemic control (3). TIND is characterized by the onset of neuropathic symptoms within 8 weeks of a ≥2% decrease in HbA1c, accompanied by objective neurological deficits (4, 5). The hallmark features of TIND include acute-onset severe neuropathic pain and/or autonomic dysfunction, with predominant involvement of small fibers (5). The exact pathophysiological mechanisms of TIND remain incompletely understood; however, it is believed to be associated with abrupt fluctuations in blood glucose levels and maladaptive neural responses. Chronic inflammatory demyelinating polyradiculoneuropathy (CIDP) is an autoimmune-mediated peripheral polyneuropathy that typically presents with progressive or relapsing limb weakness and sensory disturbances, with symptoms lasting for at least 8 weeks (6). The clinical presentation is highly heterogeneous, often leading to misdiagnosis. Therefore, differential diagnosis is essential, and early identification of diagnostic clues is crucial. Diagnosis relies on a thorough clinical history and neurological examination, supported by cerebrospinal fluid (CSF) analysis and electrophysiological studies for comprehensive evaluation (7).

This report describes the clinical course of a patient who initially presented with perineal pain as a symptom of TIND complicated by CIDP. There is no specific treatment for TIND, and its course typically lasts approximately six months. In this case, although muscle weakness improved following treatment for CIDP, pain relief was achieved only with continued analgesic therapy and, ultimately, surgery, consistent with the natural course of TIND. We discuss the potential link between TIND and CIDP, highlighting the importance of early recognition to improve patient outcomes and reduce the risk of misdiagnosis or delayed diagnosis.

## Case presentation

A 26-year-old male presented to the hospital with a two-month history of sudden-onset perineal pain, accompanied by limb weakness, and generalized body pain over the past month.

The patient had a 3-year history of DM. At the time of his initial presentation at a primary care clinic, he was started on oral metformin 0.5 g twice daily and dapagliflozin 10 mg once daily. However, adherence was poor, blood glucose monitoring was irregular, and dietary control was inadequate. More than two months before admission, he was hospitalized at a local hospital for poor glycemic control, with an HbA1c of 10.4% and received a diagnosis of T1DM. His glucose-lowering regimen was subsequently adjusted to insulin therapy, comprising glargine 22 IU at bedtime and lispro 6 IU before each meal. No diabetic complications, including diabetic kidney disease, diabetic retinopathy, or other complications, were noted during that hospitalization. Despite this regimen, his fasting blood glucose ranged from approximately 5.3 to 8.1 mmol/L, and postprandial glucose fluctuated between 4.6 and 10.1 mmol/L. One week after discharge—approximately two months before the current hospitalization—the patient developed perineal pain that progressively worsened, accompanied by mild limb weakness and myalgia.

One month prior to the current admission, intermittent generalized pain began, with a migratory pattern and prominent involvement of the lower extremities, progressively worsening over time. Despite adherence to prescribed therapy, including oral tramadol and gabapentin for pain and mecobalamin for neuroprotection, there was only modest symptom relief.

Upon admission to the Department of Endocrinology and Metabolism, the patient underwent relevant examinations and evaluations. Physical examination of the heart, lungs, and abdomen was unremarkable. Higher cognitive functions were intact. Muscle strength was graded 4/5 on the Medical Research Council (MRC) muscle strength grading scale in both the upper and lower limbs, with reduced muscle tone. Deep tendon reflexes were diminished in the upper and lower limbs, with no pathological reflexes or signs being elicited. There was no sensory ataxia observed. Hyperalgesia was noted in the perineal region, while small-fiber sensory testing in other areas was unremarkable. The anal reflex was diminished on the right side. The patient did not report autonomic symptoms, such as palpitations, diarrhea, or urinary difficulties. No abnormalities were observed in the sympathetic skin response (SSR) latency or amplitude in either upper or lower limbs on NCS.

Relevant investigations were completed, including measurements of immunoglobulins (serum), rheumatoid factor, antinuclear antibodies, anti-ENA antibody profile, anti-neutrophil cytoplasmic antibodies, HIV antibodies, and syphilis antibodies, all of which were unremarkable. MRI of the lumbar spine and hip joints, CT of the entire abdomen​​, duplex ultrasound of the lower extremity arteries and veins, bilateral scrotal vein ultrasound, and ophthalmologic examination also showed no significant abnormalities. Fasting plasma glucose was 8.1 mmol/L (reference range, 3.9–6.1 mmol/L), glutamic acid decarboxylase (GAD) antibody level was 111.3 IU/mL (reference range, 1.0–17.0 IU/mL), and HbA1c was 7.7%. Fasting C-peptide was 0.314 nmol/L. The C-peptide stimulation test yielded 1-hour and 2-hour values of 0.396 nmol/L and 0.202 nmol/L, respectively, and urinary ketones were negative. The patient had remained off insulin therapy for the past three years and reported no episodes of spontaneous ketosis. This may be attributed to a gradual decline in C-peptide secretion, suggesting the presence of a prolonged honeymoon phase. Based on these findings, the initial diagnosis upon admission was TIND and T1DM.

The patient was started on continuous subcutaneous insulin infusion (CSII) for glycemic control. His basal insulin requirement was set as insulin lispro delivered at 0.9 IU/h from 3:00 to 9:00, 0.7 IU/h from 9:00 to 22:00, and 0.5 IU/h from 22:00 to 3:00, administered via a basal–bolus regimen with pre-meal boluses of 5 IU. He was also advised to follow a diabetic diet. Following initiation of CSII, fasting blood glucose ranged from 5.8 to 7.7 mmol/L, and postprandial glucose ranged from 6.0 to 9.9 mmol/L. For neuroprotection, mecobalamin 500 μg three times daily, ebastine 50 mg three times daily, and alpha-lipoic acid 450 mg once daily were administered. Gabapentin 300mg three times daily, tramadol hydrochloride extended-release 100 mg at night, and intermittent intramuscular injections of buprenorphine hydrochloride were administered for pain management. Despite these interventions, the patient showed no improvement in symptoms. ​​Instead, he developed progressive muscle weakness,​​ graded 3/5 on the MRC scale in both the upper and lower limbs.

Nerve conduction studies (NCS) revealed evidence of peripheral nerve demyelination in the patient, affecting both sensory and motor fibers (**Tables 1**–**3**). Motor NCS demonstrated reduced conduction velocities in the right median, ulnar, tibial, and common peroneal nerves. Additionally, prolonged distal latencies and delayed F-waves were observed in the upper limb nerves. Sensory nerve conduction velocities (SCV) were decreased in the right median, ulnar, sural, and superficial peroneal nerves. CSF analysis revealed no white blood cells, a glucose level of 4.18 mmol/L (reference range: 2.5–4.5 mmol/L), a protein level of 1328.9 mg/L (reference range: 120–600 mg/L), and an albumin level of 511.00 mg/L (reference range: 0–350 mg/L). Immunoglobulin analysis showed CSF-IgA 15.10 mg/L (reference range: 0–5 mg/L), CSF-IgM 1.33 mg/L (reference range: 0–1.3 mg/L), and CSF-IgG 46.90 mg/L (reference range: 0–34 mg/L). Anti-GT1a IgG antibodies were positive. The CSF bacterial smear and other relevant tests were negative. In serum, anti-GT1a IgG and anti-GQ1b IgG antibodies were also positive.

**Table 1:** Nerve conduction study report (motor nerve conduction).

Nerve	Site (right)	Amplitude (mV)	Normal reference value (mV)	Latency (ms)	Normal reference-value (ms)	Distance (mm)	Conduction Velocity (m/s)	Normal reference-value (m/s)
Before treatment for CIDP								
Median Nerve	Wrist	6.1	≥4.8	4.1	≤4.0	280	48.9	≥50.0
	Elbow	6.4	Amplitude ratio (proximal/distal) ≥0.80	8.8				
Ulnar Nerve	Wrist	8.1	≥5.5	2.8	≤4.0	280	43.7	≥50.0
	Elbow	8.4	Amplitude ratio (proximal/distal) ≥0.80	9.2				
Tibial Nerve	Ankle	11.0	≥5.0	5.9	≤5.5	390	38.6	≥40.0
	Popliteal fossa	6.6	Amplitude ratio (proximal/distal) ≥0.50	16.0				
Common Peroneal Nerve	Ankle	4.1	≥2.3	5.3	≤5.5	350	38.4	≥40.0
	Tibial tuberosity	3.3	–	14.4				
After treatment for CIDP								
Median Nerve	Wrist	4.1	≥4.8	4.7	≤4.0	235	50.0	≥50.0
	Elbow	3.8	Amplitude ratio (proximal/distal) ≥0.80	9.4				
Ulnar Nerve	Wrist	6.9	≥5.5	3.2	≤4.0	265	44.1	≥50.0
	Elbow	5.9	Amplitude ratio (proximal/distal) ≥0.80	9.2				
Tibial Nerve	Ankle	9.7	≥5.0	5.0	≤5.5	455	42.5	≥40.0
	Popliteal fossa	7.8	Amplitude ratio (proximal/distal) ≥0.50	15.7				
Common Peroneal Nerve	Ankle	3.3	≥2.3	4.9	≤5.5	395	40.3	≥40.0
	Tibial tuberosity	3.2	–	14.7				

All nerve conduction studies were performed using the antidromic method.

**Table 2:** Nerve conduction study report (sensory nerve conduction).

Nerve	Site(right)	Amplitude (μV)	Normal reference-value (μV)	Latency (ms)		Segment	Distance (mm)	Conduction velocity (m/s)	Normal reference-value (m/s)
				Onset	Peak				
Before treatment for CIDP									
Median Nerve	Wrist	14.8	≥16.0	2.7	4.5	Digit I (thumb)-Wrist	120	44.1	≥50.0
Ulnar Nerve	Wrist	21.1	≥10.0	2.5	4.3	Digit V (little finger)-Wrist	120	48.0	≥50.0
Radial Nerve	Forearm	10.7	≥10.0	1.9	3.7	Anatomical snuff box – Forearm	110	57.8	≥50.0
Sural Nerve	Lower leg	6.7	≥7.0	4.3	6.6	Ankle-Lower leg	170	39.1	≥40.0
Superficial Peroneal Nerve	Ankle	3.9	≥5.0	4.6	7.4	Ankle-Lower leg	170	37.1	≥40.0
After treatment for CIDP									
Median Nerve	Wrist	14.5	≥16.0	2.5	4.4	Digit I (thumb)-Wrist	125	50.0	≥50.0
Ulnar Nerve	Wrist	9.6	≥10.0	2.5	4.8	Digit V (little finger)-Wrist	135	54.4	≥50.0
Radial Nerve	Forearm	5.7	≥10.0	1.9	3.4	Anatomical snuff box – Forearm	115	60.5	≥50.0
Sural Nerve	Lower leg	2.2	≥7.0	3.9	5.8	Ankle-Lower leg	165	42.5	≥40.0
Superficial Peroneal Nerve	Ankle	3.2	≥5.0	3.7	6.4	Ankle-Lower leg	155	42.3	≥40.0

All nerve conduction studies were performed using the antidromic method.

**Table 3:** Nerve conduction study report (F-Wave studies).

Nerve	F-Latency-min	Normal reference-value	F-Latency-number	Normal reference-value
Before treatment for CIDP				
Median Nerve	30.3	≤30.0	93.8	≥70.0
Ulnar Nerve	30.8	≤30.0	68.8	≥70.0
Tibial Nerve	58.1	≤55.0	100.0	≥70.0
After treatment for CIDP				
Median Nerve	29.0	≤30.0	100.0	≥70.0
Ulnar Nerve	29.4	≤30.0	100.0	≥70.0
Tibial Nerve	49.0	≤55.0	100.0	≥70.0

All nerve conduction studies were performed using the antidromic method.

The patient continued to receive glucose-lowering and analgesic treatment. Standard therapy with intravenous methylprednisolone (40 mg daily for two weeks) was initiated for CIDP, leading to marked improvement in muscle strength and significant enhancement in NCS findings (**Tables 1**–**3**). Upon discharge, oral methylprednisolone 40 mg daily was prescribed for two weeks as maintenance therapy, and further improvement was noted. However, during this period, the patient continued to suffer from severe perineal pain, which was refractory to conventional analgesics, including gabapentin, pregabalin, and carbamazepine. To relieve persistent pain, the patient underwent left-sided perineal nerve pulsed radiofrequency therapy, along with hyaluronic acid sodium injection into the pubic symphysis joint and intravenous methylprednisolone (40 mg). This multimodal approach resulted in a favorable response.

Therefore, in addition to TIND and T1DM, the patient was diagnosed with possible typical CIDP based on characteristic clinical features, electrophysiological evidence of demyelination, elevated CSF protein, and a pronounced objective response to immunomodulatory therapy (8). Then, the oral methylprednisolone dose was tapered by 4 mg every four weeks. At a 5-month follow-up, the patient demonstrated significant relief of pain symptoms, complete recovery of limb muscle strength (5/5 on the MRC scale), and stable glycemic control with fasting glucose maintained at approximately 7 mmol/L under treatment with glargine 22 IU at bedtime and lispro 4 IU before each meal.

## Discussion

In 2015, Gibbons and Freeman proposed clinical criteria for diagnosing TIND, which include: 1) a reduction in HbA1c of more than 2% within 3 months; 2) the onset of neuropathic pain or autonomic dysfunction within 8 weeks following the HbA1c reduction; and 3) the acute onset of severe neuropathic pain and/or autonomic dysfunction, sufficient to prompt the patient to seek medical attention (5). Taken together, the clinical presentation and diagnostic data of our patient support the diagnosis of TIND.

Both TIND and DPN may be linked to autonomic dysfunction (9). In patients with TIND, neuropathic pain is often described as symmetrical tingling or sharp stabbing pain, or dysesthetic pain, typically following the classic “stocking-and-glove” distribution, with more severe symptoms in the distal limbs. Autonomic symptoms may include syncope or orthostatic dizziness, gastrointestinal obstruction, and/or erectile dysfunction. The “neural energy crisis” hypothesis proposes that chronic hyperglycemia in diabetic patients reduces nerve blood flow and induces endothelial dysfunction, thereby creating a prolonged hypoxic microenvironment within the endoneurium. A sudden drop in glucose levels could result in relative endothelial hypoglycemia, triggering an “energy crisis” leading to TIND (10). Another contributing factor to TIND is the infiltration of macrophages in peripheral nerves, which leads to chronic inflammation driven by activated macrophages releasing pro-inflammatory cytokines (e.g., IL-1β, IL-6, TNF-α) that enhance the excitability of primary sensory neurons, ultimately resulting in neuropathic pain (10, 11). Some researchers also suggest nerve regeneration following blood glucose control may be another neuropathic pain source (12). However, the interplay between neurodegeneration and regeneration and its relationship with TIND requires further investigation.

The pathogenesis of CIDP primarily involves a coordinated immune response driven by cell-mediated mechanisms, humoral factors, and cytokines targeting the peripheral nerve myelin (6). In patients with CIDP, macrophage hyperactivation and expansion occur, accompanied by significant increases in the levels of various pro-inflammatory cytokines. Peripheral blood levels of cytokines such as TNF-α, IL-6, IL-17, and CXCL10 are notably elevated compared to healthy controls, indicating an ongoing immune-mediated inflammatory response (13).

Multiple studies suggest that the prevalence of diabetes is likely higher in patients with CIDP, indicating that diabetes may be a risk factor for the development of CIDP (14). Despite limited direct evidence linking TIND and CIDP, shared inflammatory pathways suggest a plausible interaction. Specifically, neuroinflammatory mediators released in TIND (e.g., TNF-α, IL-6) may exacerbate immune dysregulation, potentially priming the peripheral nervous system for autoimmune attacks characteristic of CIDP (15). In the present case, the patient had concomitant T1DM with poor glycemic control, resulting in prolonged chronic inflammation. Following rapid glucose control, TIND was triggered, leading to the release of neuroinflammatory mediators such as TNF-α, IL-1β, and IL-6, which in turn caused nerve damage (10). This nerve injury further stimulates macrophage infiltration and increases the number of activated T cells, thus perpetuating the inflammatory response and contributing to ongoing peripheral nerve damage (6, 16, 17). CSF analysis showed elevated protein, albumin, and immunoglobulins (IgA, IgM, IgG), with normal glucose and no white blood cells. Serum testing was positive for anti-GT1a IgG and anti-GQ1b IgG antibodies. These findings suggest the presence of CIDP-associated immune-mediated demyelination and peripheral nerve damage in the patient. While TIND primarily manifests as axonal degeneration due to microvascular ischemia, CIDP is classically characterized by macrophage-mediated demyelination. However, shared upstream mechanisms—such as cytokine-driven blood-nerve barrier (BNB) disruption—may link these distinct phenotypes. As the largest pro-inflammatory cell population in the body, macrophages disrupt the integrity of the BNB and promote inflammation by releasing cytokines and chemokines through CD4+ activated cells. In turn, these cytokines and chemokines further enhance macrophage activation, creating a feedback loop that amplifies the inflammatory response (18). For instance, TNF-α upregulation in TIND may enhance BNB permeability via downregulating tight junction proteins (e.g., claudin-5), allowing circulating autoantibodies (e.g., anti-GM1) to access peripheral nerves, a key step in CIDP pathogenesis (19). For patients with T1DM, the use of continuous glucose monitoring (CGM) can help detect glycemic fluctuations, guide insulin therapy, improve overall glucose control, and potentially reduce the risk of TIND.

Animal models’ evidence highlights autoreactive T-cell immunity’s central role in demyelinating diseases, particularly in CIDP (20, 21). Neuropathological findings indicate infiltration of CD4+ and CD8+ T cells, along with macrophages, within the endoneurium (22). T-cell activation within the endoneurium stimulates matrix metalloproteinases, increases neurovascular permeability, and facilitates the infiltration of pro-inflammatory cytokines and chemokines (23). Rapid glycemic control could trigger a cytokine surge (e.g., IL-6), promoting epitope spreading and increasing the risk of cross-reactive T-cell responses against peripheral nerve antigens. Additionally, chronic hyperglycemia exacerbates oxidative stress, leading to structural damage in nerve tissues, particularly impairing Schwann cell (SC) function (24). Chronic hyperglycemia-induced inflammation also worsens immune dysfunction and contributes to TIND-related neuropathic pain. Nocturnal aggravation of pain further disrupts sleep, amplifying fatigue and cognitive deficits (25, 26). This may contribute to the pathogenesis of CIDP in the patient.

In the most recent diagnostic guidelines for Guillain-Barré syndrome (GBS, 2023) and CIDP (2021), biomarkers such as CSF albumin are considered only supportive diagnostic tools (27, 28). Beyond CSF albumin, a study by Ivan Kmezic and colleagues provided preliminary evidence supporting beta-trace protein (BTP) levels in CSF and plasma as potential biomarkers for CIDP and GBS (29). Moreover, CSF BTP levels correlated with CSF neurofilament light chain (NfL) concentrations, suggesting a potential shared pathophysiological mechanism at the level of the nerve roots.

In addition, a research team from Spain reported that serum NfL levels significantly decreased in patients with CIDP who responded to maintenance intravenous immunoglobulin (IVIG) therapy, and sNfL levels correlated positively with disability (30). BTP and NfL may serve as promising biomarkers to aid in the early differential diagnosis of CIDP and facilitate timely, targeted treatment. Beyond corticosteroids, IVIG has demonstrated both safety and efficacy in CIDP, with a reported response rate of approximately 70% (6). Early diagnosis and intervention remain critical to minimizing long-term disability.

## Conclusion

TIND is a complex and enigmatic syndrome. It should be considered in diabetic patients with acute neuropathic symptoms, such as pain or autonomic dysfunction. Its pathogenesis may involve immune-mediated mechanisms, with a possible shared immunological basis between TIND and CIDP. Our case raises the possibility that TIND may serve as a trigger for CIDP in patients with T1DM. If TIND is accompanied by progressive and persistent weakness, clinicians should remain vigilant for the possible development of CIDP.

## Data Availability

The original contributions presented in the study are included in the article/supplementary material. Further inquiries can be directed to the corresponding author.
